# Efficacy of metformin combined with liraglutide on the glucose and lipid metabolism, vascular endothelial function, and oxidative stress of patients with T2DM and metabolic syndrome

**DOI:** 10.12669/pjms.40.1.7936

**Published:** 2024

**Authors:** Zaisheng Liu

**Affiliations:** 1 Zaisheng Liu Department of Endocrinology Caoxian People’s Hospital Cao Country, Heze City, Shandong Province 274400, P.R. China

**Keywords:** Glucose and lipid metabolism, Oxidative stress Liraglutide, Metformin, Metabolic syndrome, Type-2 diabetes, Vascular endothelial function

## Abstract

**Objective:**

This study evaluates the impact of metformin combined with liraglutide on the glucose and lipid metabolism, oxidative stress, and vascular endothelium of patients with type-2 diabetes mellitus (T2DM) and metabolic syndrome.

**Methods:**

Medical records of 78 patients with T2DM and metabolic syndrome, admitted to Caoxian People’s Hospital from July 2021 to July 2022, were retrospectively analysed. Thirty five patients were treated with metformin (control group), and 43 patients were treated with metformin combined with liraglutide (observation group). Indexes of glucose and lipid metabolism, function of vascular endothelium and the oxidative stress of both groups were compared before and after the treatment.

**Results:**

There was a significant decrease in the levels of fasting plasma glucose (FPG), Glycosylated Hemoglobin A1c (HbA1c), triglyceride (TG), total cholesterol (TC), low-density lipoprotein cholesterol (LDL-C), systolic blood pressure (SBP), diastolic blood pressure (DBP) and waist circumference in both groups three months after the treatment, These indexes were significantly lower in the observation group compared to the control group (*P*<0.05). High-density lipoprotein cholesterol (HDL-C) levels were higher in the observation group (*P*<0.05). There was a significant improvement in the levels of nitric oxide (NO), endothelin-1 (ET-1), superoxide dismutase (SOD), and malondialdehyde (MDA) after the treatment, and these indexes were markedly better in the observation group compared to the control group (P<0.05).

**Conclusions:**

Metformin combined with liraglutide treatment is associated with better outcomes than metformin alone in patients with T2DM and metabolic syndrome. Combined treatment results in improved glucose and lipid metabolism, vascular endothelial function, and oxidative stress index values.

## INTRODUCTION

Type-2 diabetes mellitus (T2DM) is a common chronic disease with an overall prevalence of 12.4% in China.^1^ Poor blood glucose control can lead to a variety of acute and chronic complications that can seriously affect the physical and mental health of patients and results in a heavy burden on patients and on the healthcare system in China and worldwide.^2^ Metabolic syndrome, a cluster of various risk factors, including hypertension, obesity, and lipid metabolism disorders, may lead to aggravation of T2DM.^3^ Patients with T2DM and metabolic syndrome have impaired glucose and lipid metabolism as well as vascular endothelial function damage.^2^,^3^ Metabolic syndrome affects nearly one-fifth of the adult population in Asia-Pacific region.^4^

Long-term high blood glucose levels impact the efficiency of the treatment, thus leading to the development of a vicious cycle.^5^,^6^ Clinical treatment of patients with T2DM and metabolic syndrome mainly includes hypoglycaemic drugs, such as metformin, and lifestyle changes. Metformin can promote insulin sensitivity, improve pancreatic islet β cells function, inhibit liver glycogen outputs, and reduce serum glucose levels. However, TD2M combined with the metabolic syndrome is challenging to treat with just one agent.^7^ Liraglutide is a glucagon like peptide-1 receptor agonist that reduces glucagon secretion, blood glucose drift and improves blood glucose levels. Liraglutide also induces β cell proliferation by inhibiting insulin secretion.^8^ Recent studies attempted to explore the efficiency of combined liraglutide and metformin treatment in obese patients with T2DM. However, the effect of the combined therapy on glucose and lipid metabolism, vascular endothelial function, and oxidative stress is still unclear.^9^,^10^ The aim of this study was to compare the clinical effect of metformin alone with the regimen of metformin combined with liraglutide in patients with T2DM and metabolic syndrome.

## METHODS

Clinical data of 78 patients with T2DM and metabolic syndrome, treated in Caoxian People’s Hospital from July 2021 to July 2022, were collected retrospectively. According to the treatment records, the patients were divided into the following two groups: the control group (n=35, patients who received oral metformin (Shanghai Squibb Pharmaceutical, H20023370), 0.5 g three times/day), and the observation group (n=43, patients who were treated with metformin and subcutaneous liraglutide injections (Novo Nordisk, Denmark, J20160037), 0.6 g once a day). All patients received the appropriate treatment for 3 months.

### Inclusion criteria:

- Patients met the diagnostic criteria for T2DM and metabolic syndrome according to the American Diabetes Association guideline.^11^
- Patients with duration of T2DM less than two years
- Patients aged 18-75 with no restriction on gender.
- Patients with complete medical records.

### Exclusion criteria:

- Patients with special diabetes subtypes.
- Patients with severe diabetes complications.
- Patients with severe organ dysfunctions or malignant tumors.
- Patients who were taking metformin, GLP-1 receptor agonist, corticosteroids, or any other diabetic medications like sulfonylureas or thiazolidinediones
- Pregnant and lactating women.

### Ethical Approval

The Medical Ethics Committee of our hospital approved this study (No. CXRMYYEC2022-024-01, Date: 2021-06).

The following relevant indicators were extracted before and after the 3-month treatment:

- Glucose and lipid metabolism indicators levels: FPG, HbA1c, TG, TC, high-density lipoprotein cholesterol (HDL-C), and low-density lipoprotein cholesterol (LDL-C). These indices were assessed using an automatic biochemical analyser (AU5800, Beckman Coulter, USA). Other physiological indicators such as systolic blood pressure (SBP), diastolic blood pressure (DBP) and waist circumference were also recorded.
- Vascular endothelial function measurements. Levels of NO were assessed using a nitrate reductase method and levels of ET-1 were measured with an enzyme-linked immunosorbent assay (ELISA). The kits were purchased from Nanjing Jiancheng Bioengineering Research Institute.
- Oxidative stress indicators levels, as measured by a xanthine oxidase method to quantify the levels of SOD and a thio-barbituric acid method to quantify the levels of MDA. The kits were purchased from Wuhan Bode Biotechnology.

### Statistical analysis

Categorical variables were expressed as n (%), and compared via Fisher’s exact or Chi-squared tests. Normally distributed continuous variables were expressed as means ± SDs (standard deviations) and compared by Student’s *t*-tests, while non-normally distributed continuous variables were expressed as median (interquartile range) and compared via Mann-Whitney *U* tests. P<0.05 was statistically significant. SPSS v26.0 (SPSS, Chicago, IL, USA) was used for all statistical analyses.

## RESULTS

Seventy eight patients were included in this study. There were 20 males and 15 females in the control group, with an average age of 49.88±7.68 years. The observation group included 22 males and 21 females with an average age of 50.58 ± 8.27 years. General characteristics of the patients, such as gender, age, course of disease, and BMI were comparable in both groups (P>0.05; Table-I). Levels of FPG, HbA1c, TG, TC, LDL-C, SBP, DBP, and waist circumference in both groups were similar before the treatment (*P*>0.05), and decreased significantly after three months of treatment (P<0.05). Post-treatment levels of FPG, HbA1c, TG, TC, LDL-C, SBP, DBP, and waist circumference were significantly lower in the observation group (P<0.05;. HDL-C levels were similar between the groups before the treatment and rose significantly after the treatment (P<0.05;. Post-treatment levels of HDL-C in the observation group were significantly higher than those in the control group (P<0.05; Table-II). Pre-treatment levels of NO, ET-1, SOD or MDA were comparable in both groups (P>0.05). After three months of the treatment, NO and SOD levels in each group were higher than before the treatment, and the levels of NO in the observation group were significantly higher than in the control group (P<0.05). Levels of ET-1 and MDA decreased after the treatment in both groups and were significantly lower in the observation group compared to the control group (P<0.05; Table-III). There were no reports of adverse drug reactions in either group during the treatment.

**Table-I:** Comparison of General Data of Two Groups.

Groups	n	Gender (men/women)	age (years)	T2DM course (year)	BMI (kg/m^2^)
Control group	35	20/15	49.88±7.68	4.00(3.00-5.00)	26.00(25.00-27.00)
Observation group	43	22/21	50.58±8.27	5.00(4.00-6.00)	26.00(24.00-27.00)
*χ^2^*/*t/Z*	-	0.278	-0.381	-1.248	-1.209
*P*	-	0.598	0.704	0.212	0.227

**Table-II:** Comparison of blood lipid metabolism indexes and basic physiological indicators between the two groups (*χ̅*±*S*).

Indexes	Time	Control group (n=35)	Observation group (n=43)	t/Z	P
FPG (mmol/L)	Pre-treatment	8.20±1.34	8.44±1.38	-0.779	0.438
	Post-treatment	6.80±1.23^a^	6.13±1.29^a^	2.321	0.023
HbA1c (%)	Pre-treatment	8.44±1.32	8.59±1.42	-0.480	0.632
	Post-treatment	6.78±1.22^a^	6.00±1.39^a^	2.573	0.012
TG (mmol/L)	Pre-treatment	2.81±0.37	2.88±0.38	-0.858	0.394
	Post-treatment	2.00(1.80-2.40)^a^	1.80(1.50-2.20)^a^	-2.253	0.024
TC (mmol/L)	Pre-treatment	6.68±0.88	6.89±0.85	-1.078	0.284
	Post-treatment	5.00(4.40-5.90)^a^	4.60(4.20-5.60)^a^	-2.087	0.037
LDL-C (mmol/L)	Pre-treatment	3.869±0.72	3.80±0.75	0.421	0.675
	Post-treatment	3.17±0.67^a^	2.75±0.69^a^	2.744	0.008
HDL-C (mmol/L)	Pre-treatment	1.00(0.80-1.30)	1.20(0.90-1.30)	-1.325	0.185
	Post-treatment	1.40(1.20-1.70)^a^	1.80(1.50-1.90)^a^	-3.458	0.001
SBP (mmHg)	Pre-treatment	131.06±11.93	132.79±9.68	-0.709	0.481
	Post-treatment	126.91±12.29^a^	119.65±10.61^a^	2.800	0.006
DBP (mmHg)	Pre-treatment	82.00(76.00-92.00)	87.00(74.00-93.00)	-0.558	0.577
	Post-treatment	78.00(72.00-88.00)^a^	75.00(65.00-80.00)^a^	-2.222	0.026
Waist circumference (cm)	Pre-treatment	125.14±11.39	123.21±13.61	0.671	0.504
	Post-treatment	119.97±10.94^a^	114.26±13.31^a^	2.041	0.045

***Note:*** compared with value before treatment,

a *P* < 0.05.

**Table-III:** Comparison of vascular endothelial function and oxidative stress indicators between the two groups (*χ̅*±*S*).

NO (umol/L)		ET-1 (pg/ml)		SOD (U/ml)		MDA (nmol/L)	

Groups (n)	Before treatment	After treatment	Before treatment	After treatment	Before treatment	After treatment	Before treatment	After treatment
Control group (n=35)	48.17±5.66	55.28±5.87	92.37±7.05	86.78±5.65	105.42±9.06	116.00(111.00-124.00)^a^	6.46±1.14	4.70(4.10-5.70)^a^
Observation group (n=43)	47.48±5.80	60.13±6.08	93.04±7.83	82.46±5.63	106.42±9.17	126.00(115.00-135.00)^a^	6.55±1.23	4.10(3.30-5.40)^a^
*t/Z*	0.523	-3.558	-0.396	-2.587	-0.476	-3.479	-0.323	-2.279
*P*	0.603	0.001	0.694	0.010	0.635	0.001	0.748	0.023

***Note:*** compared with values before treatment,

a P < 0.05.

## DISCUSSION

Our results showed that liraglutide combined with metformin is more effective than metformin alone in improving glucose and lipid metabolism, vascular endothelial function, and oxidative stress response in patients with T2DM and metabolic syndrome. Our results confirm previous study by Keskin et al^9^ and Anholm et al^12^, but are different from the conclusions of the pilot study by Liu et al^10^. Zeraattalab-Motlagh S et al.^13^ showed that the occurrence of T2DM is closely associated with dietary habits, lifestyle, and physical activity. The pathological mechanisms of T2DM include insulin secretion dysfunction, lipid metabolism disorders, and other factors that lead to the accumulation of multiple risk factors that may cause the metabolic syndrome, which in turn may aggravate insulin resistances and increases the incidence of diabetes complications. Therefore, a combination of T2DM with metabolic syndrome makes it difficult to control blood sugar. 14,15

We selected glucose and lipid metabolism (FPG, HbA1c, TG, TC), vascular endothelial function (NO, ET-1), and oxidative stress indicators (SOD, MDA) as the observation markers. FPG, HbA1c, TG, and TC are commonly used in clinical practice to assess lipid metabolism status of the patients.^16^ NO and ET-1 are synthesized by vascular endothelial cells, and serve as clinical markers of endothelial cell damage.^17^ NO is a vasodilator that effectively promotes relaxation of vascular smooth muscle, and ET-1 is an endogenous vasoconstrictor that promotes inflammatory reactions and proliferation of vascular smooth muscle cells. Tanaka A et al^18^ showed that patients with T2DM and metabolic syndrome are in a state of long-term hyperglycaemia and lipid metabolism imbalance that can lead to vascular endothelial dysfunction with abnormal vasoconstriction, vasospasms, etc. Groeneveld ON et al.^19^ also showed that patients with T2DM and metabolic syndrome are in a state of oxidative stress (as measured by levels of SOD and MDA). This, in turn, leads to further damage to the the vascular endothelium. SOD can promote the metabolism of oxygen-free radicals^20^, and MDA reflect the level of peroxidation.^20^,^21^

Our results show that a combined metformin-liraglutide regimen led to significant improvement in all indicators after three months of treatment compared to metformin treatment alone. Our findings are similar to the results of Dahl K et al,^22^ who showed that patients with T2DM and metabolic syndrome, treated with a combination of metformin and liraglutide, have better glucose and lipid metabolism, vascular endothelial function, and oxidative stress responses compared to patients treated with metformin alone.^7^,^22^

The increased efficiency of the combined treatment may be explained by the different mechanism of action of these two agents. Metformin acts by inhibiting liver gluconeogenesis, reducing liver glucose outputs, increasing insulin sensitivity in peripheral tissues, and thereby, reducing blood sugar levels.^7^ Moreover, metformin can reduce the formation of free fatty acids, promote lipoprotein activity, and regulate lipid metabolism disorders.^23^ Liraglutide, on the other hand, is a glucagon-like peptide-1 receptor agonist that simulates the physiological function of glucagon-like peptide-1 and reduces blood sugar levels, thus improving metabolic disorder, lowering blood pressure, and inhibiting inflammation.^24^ Liraglutide contains a C-16 palmitic acid that can replace lysine, and reversibly binds plasma albumin to form a slowly dissolving repository, prolonging its action time and its half-life, which can reach 13 hours. Therefore, liraglutide can effectively stabilize blood glucose levels throughout the day and prevents fluctuations in blood glucose levels.^24^,^25^ Therefore, a regimen that combines metformin and liraglutide can have a synergistic effect in patients with T2DM and metabolic syndrome, and more effectively control blood glucose level, improve metabolic state, reduce organ damage caused by hyperglycemia and dyslipidemia, protect vascular endothelium, reduce oxidative stress, and enhance the overall clinical efficiency of the treatment compared to metformin alone.^22^,^24^,^25^

### Limitations of the study

This study was retrospective in nature, with a small sample size and a short follow-up of only three months. That may make our conclusions subjective and one-sided. Further multi-centre studies with larger sample sizes and longer follow-ups are needed to confirm our results.

## CONCLUSION

Liraglutide combined with a metformin treatment can improve the effects of the treatment on glucose and lipid metabolism, vascular endothelial function, and oxidative stress indexes in patients with T2DM and metabolic syndrome. Our results may provide a reference for clinicians treating patients with T2DM and metabolic syndrome.

### Authors’ contributions:

**ZL:** Conceived and designed the study.

**ZL:** Collected the data and performed the analysis.

**ZL:** Was involved in the writing of the manuscript and is responsible for the integrity of the study.

All authors have read and approved the final manuscript.
